# Piperine Enhances Mitochondrial Biogenesis to Mitigate Stress in SH‐SY5Y Neuroblastoma Cells

**DOI:** 10.1002/fsn3.70637

**Published:** 2025-07-16

**Authors:** Nongluk Saikachain, Pawitchaya Charoensawat, Oramon Tuntoolavest, Napak Vejsureeyakul, Teeranart Kunpittaya, Wanangkan Poolsri, Titiwat Sungkaworn, Rungnapha Saeeng, Chatchai Muanprasat, Nithi Asavapanumas

**Affiliations:** ^1^ Chakri Naruebodindra Medical Institute, Faculty of Medicine Ramathibodi Hospital Mahidol University Samut Prakan Thailand; ^2^ Faculty of Medicine Ramathibodi Hospital Mahidol University Bangkok Thailand; ^3^ Department of Science, Technology and Innovation, Faculty of Science Chulabhorn Royal Academy; ^4^ Department of Chemistry, Faculty of Science Burapha University Chonburi Thailand

**Keywords:** mitochondria, mitochondrial dynamic, neurodegeneration, neuron, piperine

## Abstract

Mitochondrial dysfunction plays a crucial role in neurodegenerative disorders. Enhancing mitochondrial biogenesis is a promising therapeutic strategy for mitigating mitochondrial damage. Piperine, a bioactive alkaloid from black pepper, the fruit of *Piper nigrum* L. in the family Piperaceae, has demonstrated neuroprotective effects against mitochondrial stress. However, its effects on mitochondrial health remain unclear. This study investigated the effects of piperine on mitochondrial dynamics in SH‐SY5Y neuronal cells. Our findings suggest that piperine enhances mitochondrial biogenesis by upregulating peroxisome proliferator‐activated receptor gamma coactivator 1‐alpha (*PPARGC1A*) mRNA and translocase of outer mitochondrial membrane 20 (TOM20) protein expression. Additionally, piperine improves Ca^2+^ transport within mitochondria and boosts mitochondrial metabolic activity without significantly altering mitochondrial morphology. Furthermore, piperine prevents 6‐hydroxydopamine (6‐OHDA)‐induced cellular stress by alleviating the activation of *Homo sapiens* heat shock protein family A member 5 (*HSPA5*) and DNA damage inducible transcript 3 (*DDIT3*) mRNA expression and inhibiting the apoptotic Bcl‐2‐associated X protein (BAX) to B‐cell lymphoma 2 (Bcl‐2) pathway. Notably, this neuroprotective effect occurs independently of its antioxidative activity. Taken together, our results reveal a previously unexplored aspect of piperine's neuroprotective mechanism, highlighting its ability to enhance mitochondrial biogenesis and prevent mitochondrial stress in neuronal cells. Further studies, including in vivo investigations and long‐term assessments, are warranted to explore the therapeutic potential for mitochondrial dysfunction in the central nervous system.

## Graphical abstract

Piperine increases the expression of mitochondrial biogenesis, leading to increased mitochondrial mass, enhanced Ca^2+^ influx, and upregulation of mitochondrial metabolic enzymes. These effects help mitigate mitochondrial injury‐induced neuronal death caused by 6‐OHDA.

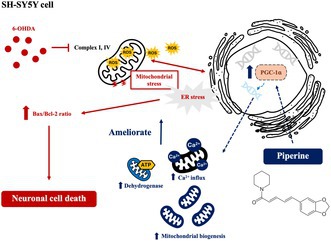

**Abbreviations**
6‐OHDA: 6‐hydroxy dopamine
BiP: binding immunoglobulin protein
CAT: catalase
CHOP: c/EBP‐homologous protein
DDIT3: DNA damage inducible transcript 3
DMSO: dimethyl sulfoxide
ER: endoplasmic reticulum
GAPDH: glyceraldehyde‐3‐phosphate dehydrogenase
GPX1: glutathione peroxidase 1
H_2_O_2_
: hydrogen peroxide
HBSS: Hanks' balanced salt solution
HSPA5:
*Homo sapiens* heat shock protein family A member 5
MFN2: mitofusin 2
MPTP: 1‐methyl‐4‐phenyl‐1,2,3,6‐tetrahydropyridine
MTT: 3‐ (4,5‐dimethylthiazol‐2‐yl)‐2,5‐ diphenyltetrazolium bromide
NAC: N‐acetylcysteine
OPA1: optic atrophy 1
Oxo‐M: oxotremorine‐M
p62: sequestosome‐1
p‐Drp1: phosphorylation of dynamin related protein 1
PGC‐1α: peroxisome proliferator‐activated receptor gamma coactivator‐1alpha
PINK1: PTEN induced kinase 1
PRKN: parkin RBR E3 ubiquitin protein ligase
RIPA: radioimmunoprecipitation assay
ROS: reactive oxygen species
SDS‐PAGE: sodium dodecyl‐sulfate polyacrylamide gel electrophoresis
SOD1: superoxide dismutase 1
TMRM: tetramethylrhodamine, methyl ester
TOM20: translocase of outer mitochondrial membrane 20

## 1 Introduction

Mitochondria are highly dynamic organelles essential for maintaining cellular homeostasis, acting as the central hubs of energy production, redox regulation, and cell fate decisions through processes such as oxidative phosphorylation, calcium signaling, and apoptosis regulation (Chen et al. 2023; Mookerjee et al. 2015; Osellame et al. 2012; San‐Millan 2023). Under physiological conditions, mitochondria have the capacity to adapt to different cellular states (Giacomello et al. 2020). Their dynamics, including coordinated processes of fission and fusion, are tightly regulated to maintain mitochondrial morphology, number, and functional capacity in accordance with the cell's metabolic demands and nutrient availability (Gomes et al. 2011a; Liesa and Shirihai 2013; Pernas and Scorrano 2016). Under nutrient‐deprived conditions, mitochondria undergo elongation, a process that enhances ATP production efficiency and protects mitochondria from autophagic degradation. This morphological adaptation is mediated by protein kinase A (PKA)‐dependent phosphorylation of DRP1, which inhibits mitochondrial fission and promotes fusion, thereby sustaining mitochondrial function and cell viability during starvation. Such remodeling not only ensures energy preservation under stress but also exemplifies the role of mitochondrial dynamics as a critical regulator of cellular fate during autophagy (Fu et al. 2019; Gomes et al. 2011b; Ren et al. 2020). Mitochondrial dynamics involve several key mechanisms, including biogenesis, fission, fusion, and mitophagy. These processes are essential for mitochondrial quality control, efficient energy production, and the maintenance of intracellular Ca^2+^ homeostasis, which are critical for supporting cell proliferation, differentiation, or triggering apoptosis (Ishihara et al. 2015; Scorrano et al. 2002; Zhao and Sheng 2024). Disruption or alterations in mitochondrial dynamics are implicated in the pathogenesis of several diseases and pathological conditions, including aging, cancer, cardiovascular disease, metabolic diseases, and neurodegenerative diseases (Bhatti et al. 2017; Chen et al. 2023; Suarez‐Rivero et al. 2016; Sukhorukov et al. 2024; Zong et al. 2024).

The central nervous system has a high energy demand to maintain ion homeostasis and perform essential functions. Parkinson's disease is an age‐related disorder characterized by the aggregation of misfolded alpha‐synuclein protein into Lewy bodies and the loss of dopaminergic neurons in the substantia nigra pars compacta (Poewe et al. 2017). This region plays a critical role in motor function, and its degeneration results in the clinical symptoms observed in Parkinson's disease patients, such as resting tremor, bradykinesia, and rigidity (Calabresi et al. 2023; Mehra et al. 2019; Poewe et al. 2017). In addition to protein aggregation, decreased mitochondrial enzymatic activity and abnormal oxidation have been identified in both in vitro and in vivo models of Parkinson's disease (Hattori and Sato 2024; Henrich et al. 2023). Furthermore, genetic variants affecting mitochondrial dynamics, such as mutations in PTEN‐induced kinase 1 (PINK1), Parkin, and DJ‐1, are associated with early‐onset forms of Parkinson's disease (Barazzuol et al. 2020; Brooks et al. 2009; Cookson 2012; Ge et al. 2020). Since a dysregulation of mitochondrial dynamics involves the pathological condition, enhancing mitochondrial dynamics is shown to have a beneficial effect for health (Chen et al. 2023; Henrich et al. 2023; Picca et al. 2018). Several approaches such as exercise and dietary control are shown to improve mitochondrial dynamics (Kyriazis et al. 2022; Sorriento et al. 2021). One of the key mechanisms involves activation of AMP‐activated protein kinase (AMPK), which in turn stimulates the expression of peroxisome proliferator‐activated receptor gamma coactivator‐1 alpha (PGC‐1α), a master regulator of mitochondrial biogenesis (Campos et al. 2023; Fernandez‐Marcos and Auwerx 2011; Herzig and Shaw 2018; Jager et al. 2007; Scalzo et al. 2014). In addition to exercise, several compounds have been identified to alleviate neurodegenerative diseases, such as inhibiting mitochondrial fission by targeting Dynamin‐related protein 1 (Drp‐1), which improves the pathology in Alzheimer and Parkinson disease (Cai et al. 2024; Cui et al. 2010; Fan et al. 2024; Masaldan et al. 2022; Oliver and Reddy 2019; Rahmani et al. 2023; Wang et al. 2017). While a growing number of compounds have been reported to support mitochondrial function—through mechanisms such as enhancing oxidative phosphorylation, stimulating mitochondrial biogenesis, or attenuating oxidative stress—the translation of these findings into approved clinical therapies remains limited. Agents like idebenone, imeglimin, and mitochondrial pyruvate carrier inhibitors have demonstrated encouraging effects in preclinical models and early‐phase clinical trials, particularly in the context of mitochondrial and metabolic disorders (Andreux et al. 2013; Singh et al. 2021; Weissig 2020). However, despite this progress, most of these compounds still require validation in large‐scale clinical studies before they can be widely adopted in clinical practice.

Xenobiotic and functional foods are emerging trends for improving individual health. Piperine, a bioactive alkaloid compound extracted from black peppers, is commonly used as a seasoning in daily food and in folk medicine (Quijia et al. 2021; Wattanathorn et al. 2008; Zhang et al. 2021). Piperine has demonstrated numerous health benefits through mechanisms such as antioxidant activity, anti‐apoptotic properties, enhancing autophagic activity, and the prevention of mitochondrial damage in the cardiovascular and central nervous system (CNS) (Haq et al. 2021; Hua et al. 2019; Kaushik et al. 2021; Ren and Zuo 2019; Saini et al. 2023; Vijayakumar et al. 2004; Viswanadha et al. 2021; Yang et al. 2015). In the context of CNS, piperine and its derivatives have shown potential in preventing neuronal injury caused by mitochondrial toxins, including 6‐hydroxydopamine (6‐OHDA), 1‐methyl‐4‐phenyl‐1,2,3,6‐tetrahydropyridine (MPTP) and rotenone compounds widely used in Parkinson's disease models (Bi et al. 2015; Liu et al. 2016; Wang et al. 2016; Yu et al. 2024). Specifically, Liu et al. reported that piperine prevents neuronal loss by activating autophagy in rotenone‐induced in vivo and in vitro models (Liu et al. 2016). Similarly, Yu et al. observed increased autophagy in 6‐OHDA‐treated rats following piperine administration (Yu et al. 2024). In contrast, Bi et al. demonstrated that piperine enhances antioxidation properties to prevent the loss of tyrosine hydroxylase‐positive neurons in an MPTP‐induced mouse model of Parkinson's disease (Bi et al. 2015). Although evidence supports piperine's protective effects against mitochondrial damage, the precise mechanisms underlying its impact on mitochondrial health remain unclear.

Therefore, in this present study, we aim to investigate the effects of piperine on mitochondrial health, including its biogenesis, morphology, and mitochondrial Ca^2+^ transport in neuronal cells using the SH‐SY5Y human neuroblastoma cell line. Furthermore, we examined whether these effects contribute to piperine's neuroprotective role in mitigating 6‐OHDA‐induced mitochondrial injury.

## 2 Materials and Methods

### 2.1 Cell Culture

SH‐SY5Y, human neuroblastoma cells (CRL‐2266, ATCC, USA) were cultured in Dulbecco's Modified Eagle Medium/Nutrient Mixture F‐12 (DMEM/F‐12) (Gibco, USA) with 10% fetal bovine serum (Sigma‐Aldrich, USA) and 1% penicillin/streptomycin (Invitrogen, USA). The SH‐SY5Y cell line is not listed as a commonly misidentified cell line by the International Cell Line Authentication Committee. STR analysis was performed for cell line authentication (ATCC, Lot number 70027507). When cells reached 90%–100% confluency, they were detached using 0.05% trypsin (Gibco, USA) and subcultured to maintain the cell line. Cells were maintained in a 5% CO_2_ atmosphere at 37°C. All the experiments were performed in SH‐SY5Y cells at passages 41–50.

### 2.2 Cell Viability Assay

SH‐SY5Y cells were seeded on a 96‐well plate at 25,000 cells/well in DMEM/F‐12 with the supplement for 24 h. Cells were pretreated with piperine (purity > 98%, prepared as previously described (Raman and Gaikar 2002); HPLC confirmation shown in Data S1) for 24 h in DMEM/F‐12 with supplements. Piperine was dissolved in DMSO before being added to the culture medium. The final DMSO concentration in all piperine‐treated groups was kept at 0.4%. To control for any effects of the solvent, we included a vehicle control group treated with 0.4% DMSO alone in all experiments. N‐acetylcysteine (NAC) was used for positive control. After that, cells were treated with 6‐OHDA for 24 h. Cell viability was measured with the 3‐(4,5‐dimethylthiazol‐2‐yl)‐2,5‐diphenyltetrazolium bromide (MTT, 298‐93‐1, Bio Basic Inc., Canada). A 100 μL MTT solution (0.5 mg/mL) was added to each well and incubated at 37°C for 2 h. After incubation, 100 μL of DMSO (CARLO ERBA, France) was added to dissolve formazan and measure absorbance at 570 nm. The concentration of 6‐OHDA toxicity was nonlinearly fitted, and the IC₅₀ of 6‐OHDA was calculated by *Y* = 100/(1 + (IC₅₀/X)^HillSlope^). For the hydrogen peroxide (H_2_O_2_) experiment, the concentration of H_2_O_2_ 100 μM was selected based on dose–response experiments, which identified an IC₅₀ of approximately 105.5 μM (Data S2); this sub‐lethal dose consistently reduced cell viability and was therefore used to assess potential protective effects. For cell proliferation analysis, we followed the protocol provided in the EdU Assay Kit (iFluor 488; Abcam, ab219801), using complete medium with serum as a positive control.

### 2.3 Mitochondrial Superoxide Measurement

Mitochondrial ROS production was determined by using a mitochondrial O_2_˙ molecular sensor (MitoSOX Red, M36008, Invitrogen, USA). The SH‐SY5Y cells were pretreated with 5, 10, and 20 μM of piperine for 24 h, followed by cotreatment with 6‐OHDA for 6 h. After that, cells were stained with 5 μM of MitoSOX prepared in Hanks' Balanced Salt Solution (HBSS) and incubated for 30 min at 37°C in the dark. After incubation, the stained solution was removed and replaced by HBSS. The MitoSOX signal was determined through excitation and emission wavelengths of 510 and 590 nm.

### 2.4 Mitochondrial Membrane Potential

Mitochondrial membrane potential was determined by Tetramethylrhodamine, methyl ester (TMRM) (1IVM‐T668, Invitrogen, USA). Cells were pretreated with piperine for 24 h and co‐treated with 6‐OHDA for 24 h. After that, cells were stained with TMRM solution in HBSS for 30 min at 37°C in the dark. After incubation, HBSS was replaced. The signal of TMRM was determined through excitation and emission wavelengths of 548 and 574 nm.

### 2.5 Mitochondrial Calcium Measurements

The mitochondrial calcium imaging was performed as described previously (Chaiwijit et al. 2023). Briefly, SH‐SY5Y cells were incubated with 1 μM Rhod2‐AM in an external solution (145 mM NaCl, 2 mM KCl, 5 mM NaHCO_3_, 1 mM MgCl_2_, 2.5 mM CaCl_2_, 10 mM glucose, and 10 mM Na‐HEPES, pH 7.25) at 37°C for 15 min. Then, the cells were washed with the external solution for 5 min at 37°C. Rhod2 fluorescence signal was time‐lapse imaged at 581 nm at 37°C using the Zeiss LSM 900 confocal laser scanning microscope (Carl Zeiss, Jena, Germany). To trigger Ca^2+^ release from ER stores through IP3 receptors (IP3Rs), 100 μM Oxotremorine‐M (Oxo‐M) was added to the imaging chamber for 2 min. The dynamics of mitochondrial Ca^2+^ levels were calculated as the relative differences in Rhod2 fluorescence intensity compared to baseline fluorescence (control).

For the mitochondrial length analysis, cells were incubated with MitoTracker (Thermo Fisher) and Hoeschst (1:500, Invitrogen (H3570)) in HBSS for 30 min at 37°C. The live‐cell imaging was performed using the Zeiss LSM 900 confocal laser scanning microscope (Carl Zeiss, Jena, Germany) at 37°C with 5% CO_2_. Mitochondrial morphology analysis was conducted using the Mitochondrial Network Analysis (MiNA) toolset (Valente et al. 2017) add‐on package in ImageJ software. A schematic representation of the analytical workflow is shown in Figure 2A. To prevent noise interference, particles with diameters smaller than 0.1 μm were excluded from the analysis after skeletonization.

### 2.6 Gene Expression

Total RNA from SH‐SY5Y cells was extracted using the Total RNA Isolation Kit (RB050, Geneaid). The concentration and quality of the RNA were assessed using a NanoDrop spectrophotometer. After passing quality control, the RNA was reverse transcribed into cDNA using the iScript Reverse Transcription Supermix (Bio‐Rad, California), following the manufacturer's instructions. Gene expression levels were quantified by quantitative PCR (qPCR) using the iTaq Universal SYBR Green Supermix (Bio‐Rad, California). The thermal cycling protocol was programmed according to the manufacturer's guidelines, consisting of an initial polymerase activation and DNA denaturation step at 95°C for 30 s, followed by 40 cycles of denaturation at 95°C for 5 s and annealing/extension at 60°C for 30 s. Melt‐curve analysis was performed from 65°C to 95°C with 0.5°C increments every 2–5 s per step. Data analysis was conducted using Bio‐Rad CFX Maestro software, and GAPDH mRNA expression was used as the internal control. The primer sequences used in this study are listed in Table 1.

**TABLE 1:** List of the primers used for real time PCR.

Gene name	NM	Targeted protein	Forward primer sequence (5′–3′)	Reverse primer sequence (5′–3′)
DNA Damage Inducible Transcript 3 (*DDIT3*)	NM_001195053.1	C/EBP homologous protein (CHOP)	AGCGACAGAGCCAAAATCAG	TCTGCTTTCAGGTGTGGTGA
*Homo sapiens* heat shock protein family A member 5 (*HSPA5*)	NM_005347.5	Binding immunoglobulin protein (BiP)	CATCAAGTTCTTGCCGTTCA	ATGTCTTTGTTTGCCCACCT
PTEN induced kinase 1 (*PINK1*)	NM_032409.3	PINK1	GGACGCTGTTCCTCGTTA	ATCTGCGATCACCAGCCA
Parkin RBR E3 ubiquitin protein ligase (*PRKN*)	NM_004562.3	Parkin	CTGACACCAGCATCTTCCAG	CCAGTCATTCCTCAGCTCCT
Sequestosome 1 (*SQSTM1*)	NM_003900.5	p62	AAATGGGTCCACCAGGAAACTGGA	TCAACTTCAATGCCCAGAGGGCTA
Peroxisome proliferator‐activated receptor gamma coactivator 1 alpha (*PPARGC1A*)	NM_001330751.2	PGC‐1α	TCAGTCCTCACTGGTGGACA	TGCTTCGTCGTCAAAAACAG
Glutathione peroxidase 1 (*GPX1*)	NM_000581.4	GPX1	CAACCAGTTTGGGCATCAG	CACCGTTCACCTCGCAC
Superoxide dismutase 1 (*SOD1*)	NM_000454.5	SOD1	ATCCTCTATCCAGAAAACACG	ACACCACAAGCCAAACGAC
Catalase (*CAT*)	NM_001752.4	CAT	TGTTGAAGATGCGGCGAG	ATGAGAGGGTAGTCCTTGTG
Glyceraldehyde‐3‐phosphate dehydrogenase (*GAPDH*)	NM_002046.7	GAPDH	GAAATCCCATCACCATCTTCC	AAATGAGCCCCAGCC TTCTC

### 2.7 Western Blot

Total protein was extracted from SH‐SY5Y cells by RIPA lysis buffer (Thermo scientific, USA) with phosphatase inhibitor (Roche, Germany) and protease inhibitor (Roche, Germany). Protein concentration was measured by the Pierce BCA assay kit (Thermo scientific, USA). Proteins were separated by SDS‐PAGE using a 15% polyacrylamide gel (Bio‐Rad, USA). Proteins were blotted onto nitrocellulose membranes and blocked with 5% skim milk (HIMEDIA, India). After that, the membranes were incubated with primary antibodies (Cell signaling) ratio of 1:1000, Bax, Bcl‐2, OPA1, Drp1, Mfn2, TOM20, and β‐actin at 4°C overnight. The membranes were washed and incubated with horseradish peroxidase (HRP)‐conjugated secondary antibodies (Abcam) ratio of 1:20,000 at room temperature for 1 h. After incubating with HRP substrates (ECL detection kit, Merck, Germany), the membranes were then exposed in the Bio‐Rad ChemiDoc system, and the analyses were performed using Image Lab software.

### 2.8 Statistical Analysis

GraphPad Prism 10 software (GraphPad Software Inc) was used for statistical analysis in this study. Sample size was estimated based on previous studies on neuroblastoma cell lines (He et al. 2018; Saikachain et al. 2023) with an assumption of power of 0.8 and a type I error rate of 0.05. All data were presented as Mean ± SD. The Shapiro–Wilk test was used for accessing the normality distribution of each data set. No test for outliers was conducted in this study. An unpaired Student's *t*‐test was performed to compare two independent variables for the normal distribution data set. The Mann–Whitney test was performed to compare two independent variables for the non‐normal distribution data set. A one‐way ANOVA test, followed by Dunnett's post hoc test, was performed for comparisons among several groups.

## 3 Results

### 3.1 Piperine Enhances Mitochondrial Biogenesis in SH‐SY5Y Cells

**FIGURE 1:**
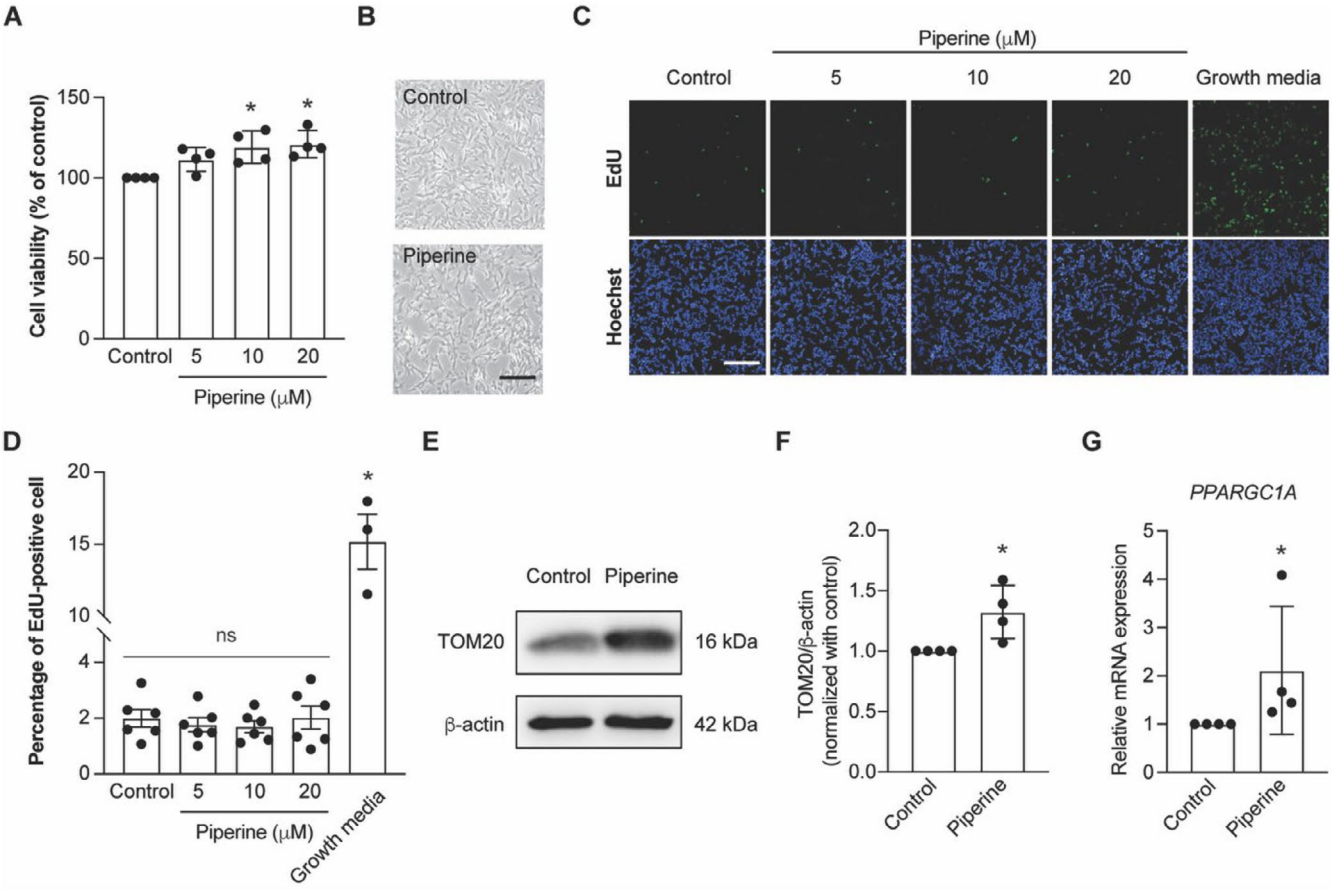
Piperine enhances mitochondrial biogenesis. (A) Bar graph represents the percentage of SHSY‐5Y cell viability compared to the control after incubating with piperine at the concentrations 5, 10, and 20 μM for 24 h across independent experiments. *p < 0.05 compared with the control group (one‐way ANOVA, *n* = 4 independent cell culture preparations). (B) Bright field images represent SHSY‐5Y morphology after incubating with 0 (control) and 20 μM of piperine for 24 h. (C) Fluorescent images representing an EdU and Hoechst staining of SH‐SY5Y cells in control, piperine at concentrations of 5, 10, 20 μM, and Growth media. (D) Bar graph represents a percentage of EdU‐positive cell in control, piperine at concentrations of 5, 10, 20 μM, and Growth media. *p < 0.05 compared with control group, one‐way ANOVA, *n* = 6 independent cell culture preparations. (E) Representative of TOM20 and β‐actin protein expression by Western blot analysis in control and piperine‐treated groups. (F) Bar graph represents normalized densitometric quantification of TOM20 relative to β‐actin, expressed as a fold change compared to the control, in both control and 20 μM of piperine‐treated groups across independent experiments. *p < 0.05 compared with control group, student's test, *n* = 4 independent cell culture preparations. (G) Bar graph represents normalized mRNA expression levels of *PPARGC1A*, expressed as a fold change compared to the control, in both control and 20 μM of piperine‐treated groups across independent experiments. *p < 0.05 compared with control group, Mann–Whitney test, *n* = 4 independent cell culture preparations.

To evaluate the neuroprotective effect of piperine, we first assessed its cytotoxicity in SH‐SY5Y cells. The cells were incubated with piperine at a concentration ranging from 0 to 80 μM for 24 h. Piperine at a concentration up to 40 μM did not reduce cell viability, indicating that it is non‐toxic to SH‐SY5Y cells at the low concentration (Data S3). Interestingly, piperine increases the MTT values, with a maximum effect observed at 20 μM (Data S3, Figure 1A), even though cell density remained comparable to the control group (Figure 1A,B). Therefore, piperine at the concentration up to 20 μM was selected for subsequent experiments to evaluate its mechanism of action. To determine whether this increase in MTT signal was due to enhanced proliferation, we performed EdU staining following 24‐h piperine treatment. The results showed no significant change in cell proliferation (Figure 1C,D), confirming that the elevated MTT signal is not due to increased cell number. This observation suggests that piperine may enhance mitochondrial function. To further investigate mitochondrial function, we assessed the active mitochondrial mass by measuring the expression of TOM20 protein (Park et al. 2019). Piperine at a concentration of 20 μM significantly increased the expression level of TOM20 protein (Figure 1E,F). Additionally, the mRNA expression of a key regulator of mitochondrial biogenesis, *PPARGC1A*, was also significantly upregulated by piperine (Figure 1F).

Taken together, these results suggest that piperine at a concentration of 20 μM enhances mitochondrial biogenesis, mass, and activity in the neuroblastoma cell line.

### 3.2 The Effect of Piperine on Mitochondrial Dynamics

**FIGURE 2:**
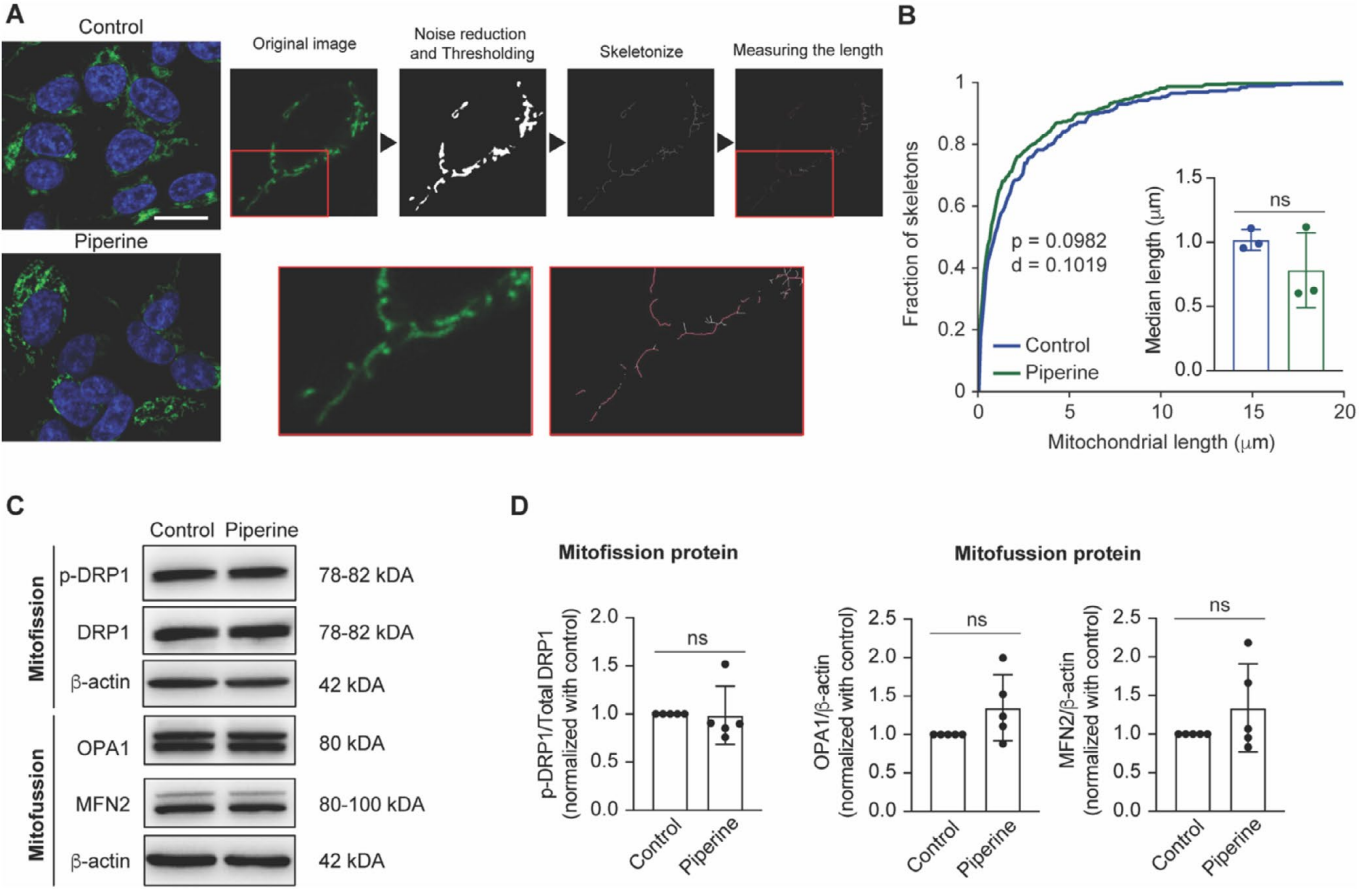
Morphological analysis of mitochondria in SH‐SY5Y cells treated with piperine. (A) Schematic representation of morphology analysis of mitochondria using the Mitochondrial Network Analysis tool set (Valente et al. 2017). (B) Cumulative distribution of mitochondrial length in control (blue) and 20 μM piperine‐treated (green) groups. Two‐sample Kolmogorov–Smirnov test: *D* = 0.10, *p* = 0.10 for control (*n* = 258 mitochondrial object) vs. piperine (*n* = 318 mitochondrial object). (Inset) Bar graph representing the median of mitochondrial length in control (blue) and 20 μM piperine‐treated (green) groups. Student's *t*‐test *p* = 0.25, *n* = 3 independent cell culture preparations. (C) Representative of p‐DRP1, DRP1, OPA1, MFN2 and β‐actin protein expression by Western blot analysis in control and 20 μM piperine‐treated groups. (D) Bar graph represents normalized densitometric quantification of p‐DRP1 to total DRP1 (Mann–Whitney, *p* = 0.14, *n* = 5), OPA1 to β‐actin (Mann–Whitney, *p* = 0.13, *n* = 5) and MFN2 to β‐actin (Mann–Whitney, *p* = 0.64, *n* = 5 independent cell culture preparations), expressed as a fold change compared to the control, in both control and 20 μM of piperine‐treated groups across independent experiments.

Besides mitochondria mass, mitochondria morphology and length also play a crucial role in maintaining cellular energy balance. Therefore, we compared the morphology of mitochondria between the control and the piperine‐treated group. Mitochondria were labeled using Mitotracker dye. Figure 2A illustrates the analytical workflow for examining mitochondria length. Although piperine treatment significantly increased TOM20 protein expression the distribution of the mitochondria lengths in the piperine‐treated group shifted slightly toward a shorter value (Figure 2B). Despite this shift, there is no significant difference in the median of the mitochondrial length between the control and piperine‐treated groups (Figure 2B, inset). This suggests that piperine treatment does not induce a global change in mitochondrial length. To further evaluate the mitochondrial morphology, we assessed the expression of the key mitochondrial fission (DRP1) and fusion proteins (OPA1 and MFN2) between the control and piperine‐treated groups. The expression level of the phosphorylated DPR1 (p‐DRP1) protein was unchanged in the piperine‐treated group compared to the control group. Although there was a trend toward increased OPA1 and MFN2 protein expression in the piperine‐treated group, this did not reach statistical significance (Figure 2C,D).

Altogether, these results indicate that while piperine increases mitochondrial mass, it does not induce global changes in mitochondrial morphology nor significantly alter mitochondrial fission and fusion processes.

### 3.3 The Effect of Piperine on Calcium Transport Into Mitochondria via Mitochondria‐Associated Membranes

**FIGURE 3:**
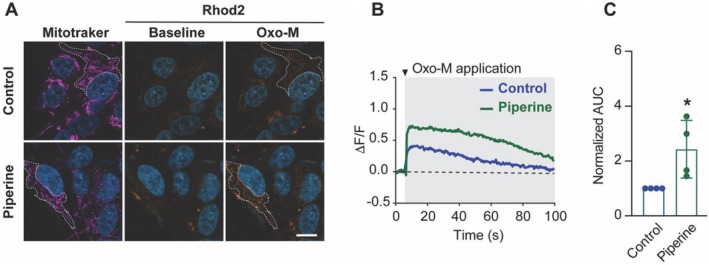
Enhanced Oxo‐M induced Ca^2+^ transfer to mitochondria in piperine‐treated SH‐SY5Y cells. (A) Maximum intensity projection (MIP) images of Mitotracker‐ and Rhod‐2‐loaded SH‐SY5Y cells, showing control and 20 μM piperine‐treated groups at the baseline (before Oxo‐M application) and during Oxo‐M application. (B) Representative traces of Rhod‐2 fluorescence intensity from (A) recorded during Oxo‐M application (indicated by tick mark). (C) Bar graph represents average area under the curve (AUC) of Rhod‐2 fluorescence intensity per cell during Oxo‐M application, expressed as a fold change compared to the control, in both control and 20 μM of piperine‐treated groups across independent experiments. *p < 0.05 compared with a control group, Student's *t*‐test, *n* = 4 independent cell culture preparations.

Mitochondrial functions, including ATP synthesis, are tightly regulated by mitochondrial Ca^2+^ homeostasis (Bravo et al. 2011; Fink et al. 2017). To investigate the effect of piperine on mitochondrial Ca^2+^ homeostasis, we used Rhod‐2 dye, a fluorescent calcium indicator dye specifically trapped within the mitochondrial matrix. Mitotracker dye was employed to confirm the localization of the Rhod‐2 signal to the mitochondrial compartment (Figure 3A). Mitochondrial Ca^2+^ influx via mitochondria‐associated membranes, a well‐characterized pathway for mitochondrial Ca^2+^ transfer (Krols et al. 2016; Loncke et al. 2021), was measured following induction with oxo‐M, a muscarinic acetylcholine receptor agonist (Figure 3B). Interestingly, the piperine‐treated group exhibited a significant increase in mitochondrial Ca^2+^ influx compared to the control group (Figure 3C).

This finding demonstrates that piperine significantly influences mitochondrial function by increasing Ca^2+^ influx via mitochondria‐associated membranes.

### 3.4 Piperine Protects SH‐SY5Y Cells From Mitochondrial Stress‐Induced Cell Death

**FIGURE 4:**
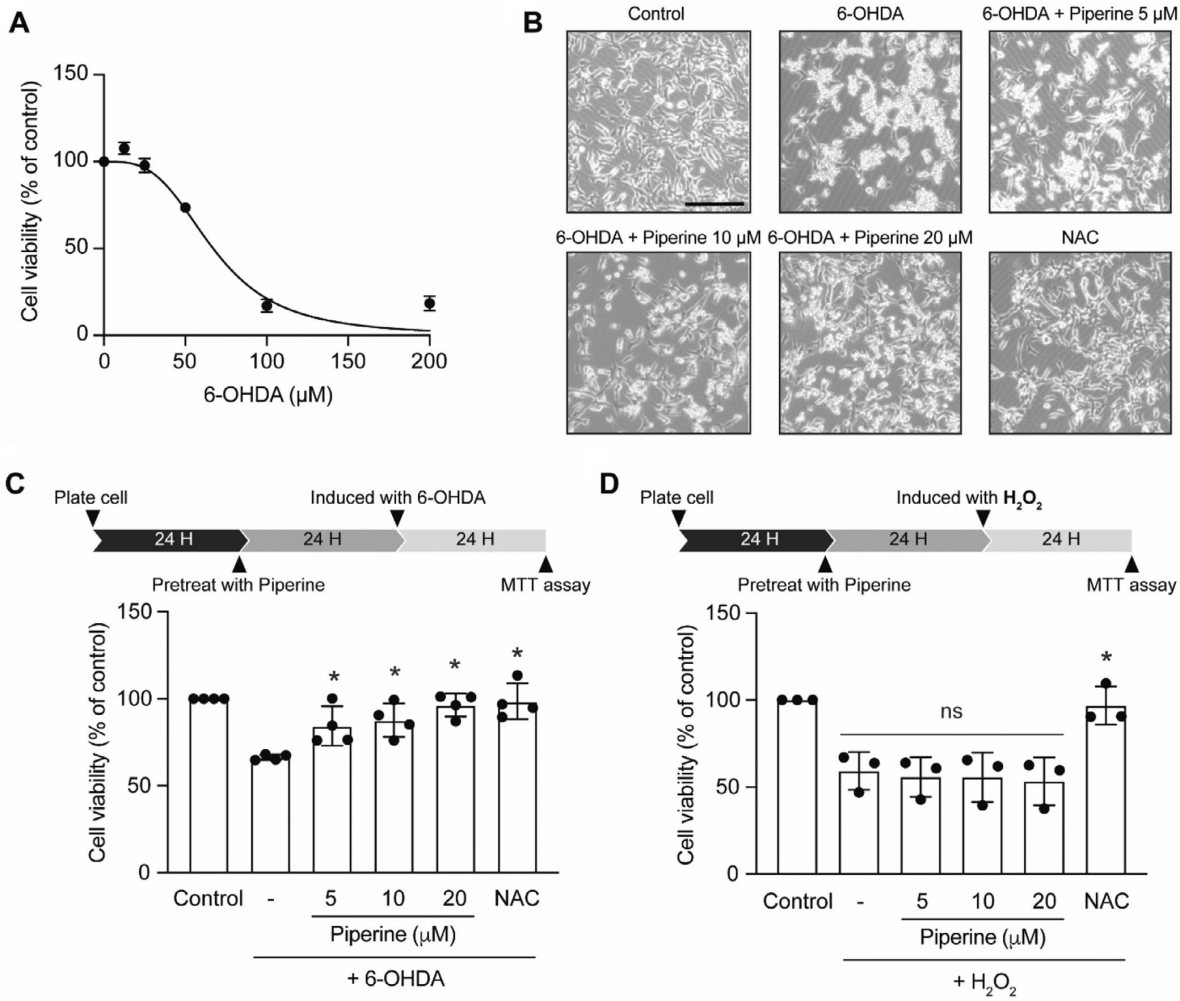
Piperine prevents 6‐OHDA‐induced‐mitochondrial injury in SH‐SY5Y cells. (A) Cell viability assay in SH‐SY5Y cells treated with 6‐OHDA at concentrations ranging from 0 to 200 μM for 24 h. Cell viability was measured using the MTT assay. Data are representative of 3 independent cell culture preparations. (B) Bright‐field images showing SH‐SY5Y cell morphology in the following groups: control, 6‐OHDA, 6‐OHDA with piperine at concentrations of 5, 10, 20 μM, and 6‐OHDA + NAC. (C) Bar graph represents the percentage of SHSY‐5Y cell viability compared to the control across independent experiments in the following groups: control, 6‐OHDA, 6‐OHDA with piperine at concentrations of 5, 10, 20 μM, and 6‐OHDA + NAC groups. *p < 0.05 compared with 6‐OHDA group, one‐way ANOVA, *n* = 4 independent cell culture preparations. (D) Bar graph represents the percentage of SHSY‐5Y cell viability compared to the control across independent experiments in the following groups: control, H_2_O_2_, H_2_O_2_ with piperine at concentrations of 5, 10, 20 μM, and H_2_O_2_ + NAC groups. *p < 0.05 compared with H_2_O_2_ group, one‐way ANOVA, *n* = 3 independent cell culture preparations.

To investigate whether the increase in mitochondrial function induced by piperine provides protection against mitochondrial stress, a key factor in the pathogenesis of neurodegenerative disease. 6‐OHDA, a neurotoxin that specifically induces mitochondrial stress by inhibiting complexes I and IV. This compound is a well‐established in vitro model of Parkinson's disease. Initially, the cytotoxicity of 6‐OHDA was evaluated at various concentrations (0–200 μM) for 24 h. Figure 4A demonstrates that 6‐OHDA reduces cell viability in a dose‐dependent manner, with an IC_50_ of approximately 65 μM. Based on this result, a concentration of 65 μM 6‐OHDA for 24 h was used in the subsequent experiment. Next, we examined the protective effect of piperine on 6‐OHDA‐induced cell death. Cells were pretreated with piperine (5, 10, and 20 μM) prior to 6‐OHDA exposure. Interestingly, piperine significantly prevented 6‐OHDA‐induced cellular injury starting from 5 μM, with the maximum protection observed at 20 μM (Figure 4B,C). However, higher concentrations of piperine appeared to reduce this protective effect (Data S4). N‐acetylcysteine (NAC) was used as a positive control. To determine whether the protective effects of piperine are specific to mitochondrial oxidative stress, the whole cell's oxidative stress was induced using 100 μM of H_2_O_2_. Notably, piperine did not prevent H_2_O_2_‐induced neuronal death (Figure 4D).

Altogether, these results demonstrate that piperine significantly prevents 6‐OHDA‐induced cell death in a dose‐dependent manner. Furthermore, the protective effects of piperine are specific to mitochondrial oxidative stress and do not extend to oxidative stress induced by H_2_O_2_.

### 3.5 The Effect of Piperine on 6‐OHDA‐Induced Mitochondrial Dysfunction

**FIGURE 5:**
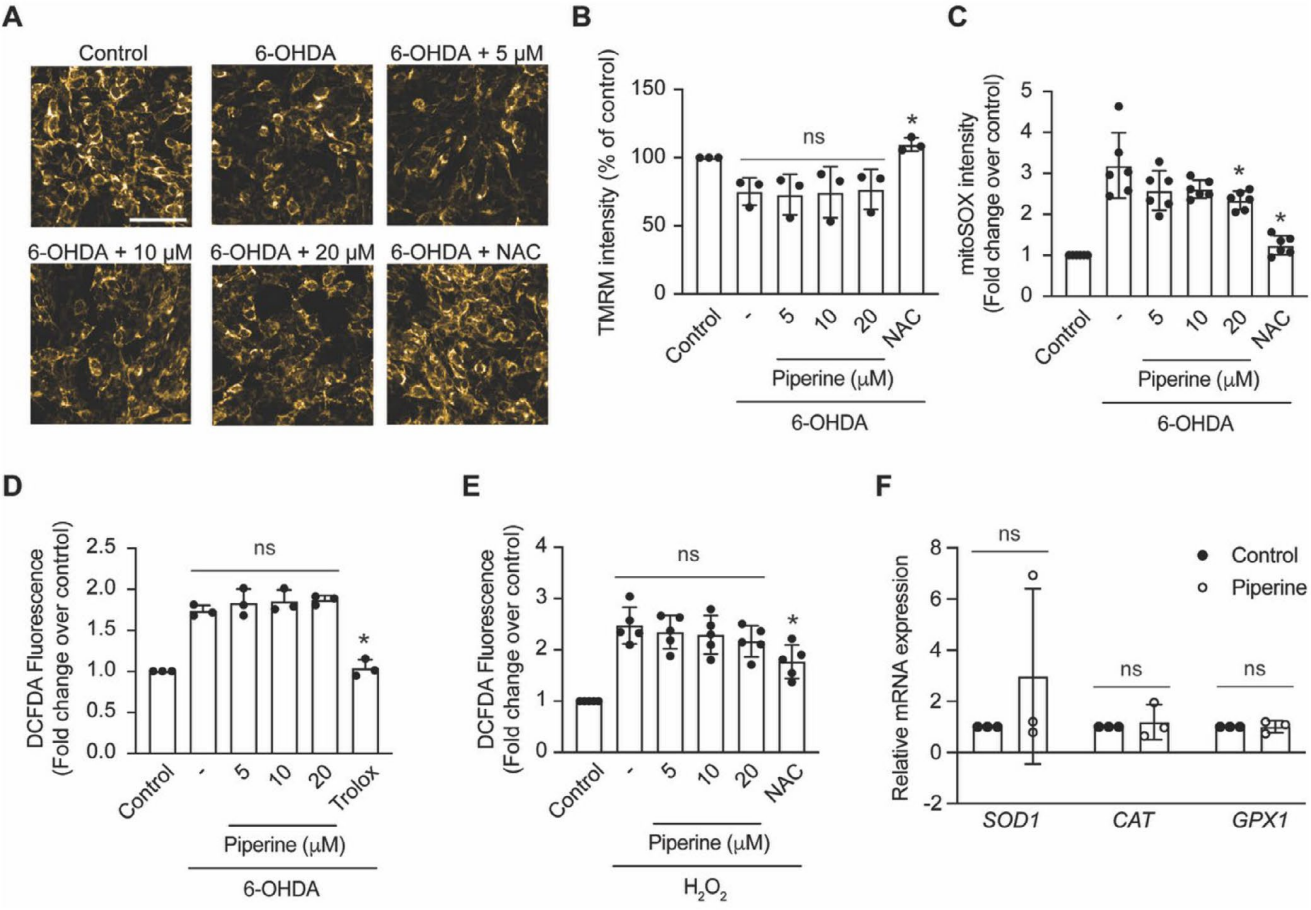
Piperine does not affect antioxidant properties in SH‐SY5Y cells. (A) Fluorescent images representing a TMRM staining of SH‐SY5Y cells in control, 6‐OHDA, 6‐OHDA with piperine at concentrations of 5, 10, 20 μM, and 6‐OHDA + NAC. (B) Bar graph represents percentage TMRM fluorescent intensity compared to the control across independent experiments in control, 6‐OHDA, 6‐OHDA with piperine at concentrations of 5, 10, 20 μM, and 6‐OHDA + NAC. *p < 0.05 compared with 6‐OHDA group, one‐way ANOVA, *n* = 3 independent cell culture preparations. (C) Bar graph represents normalized mitoSOX intensity as a fold change compared to the control across independent experiments in control, 6‐OHDA, 6‐OHDA with piperine at concentrations of 5, 10, 20 μM, and 6‐OHDA + NAC. *p < 0.05 compared with 6‐OHDA group, one‐way ANOVA, *n* = 6 independent cell culture preparations. (D) Bar graph representing a normalized DCFDA fluorescent intensity as a fold change compared to the control across independent experiments in control, 6‐OHDA, 6‐OHDA with piperine at concentrations of 5, 10, 20 μM, and 6‐OHDA + Trolox. *p < 0.05 compared with 6‐OHDA group, one‐way ANOVA, *n* = 3 independent cell culture preparations. (E) Bar graph representing a normalized DCFDA fluorescent intensity as a fold change compared to the control across independent experiments in control, H_2_O_2_ with piperine at concentrations of 5, 10, 20 μM, and H_2_O_2_ + NAC. *p < 0.05 compared with H_2_O_2_ group, one‐way ANOVA, *n* = 5 independent cell culture preparations. (F) Bar graph representing the relative mRNA expression levels as a fold change compared to the control across independent experiments of *SOD1*, *CAT* and *GPX1* in control and 20 μM piperine‐treated group (student's test, *n* = 3 independent cell culture preparations).

The mechanism of action of 6‐OHDA involves the inhibition of respiratory enzymes leading to mitochondrial dysfunction, loss of mitochondrial membrane potential, increase of intracellular ROS, and induce cell apoptosis (Glinka and Youdim 1995). To investigate how piperine prevents the cytotoxicity of 6‐OHDA in SH‐SY5Y cells, the mitochondrial membrane potential was first measured using TMRM dye. As expected, the 6‐OHDA‐treated group exhibited a significant decrease in TMRM fluorescent intensity compared to the control group. However, piperine did not reverse the decrease in TMRM fluorescence caused by 6‐OHDA (Figure 5A,B). Next, mitochondrial superoxide accumulation was evaluated using MitoSOX indicator dye. The 6‐OHDA‐treated group showed a significant increase in mitochondrial superoxide level, while the piperine‐treated group at a concentration of 20 μM exhibited a slight but significant reduction in the MitoSOX intensity induced by 6‐OHDA (Figure 5C).

To determine whether the reduction in mitochondrial superoxide by piperine was due to its antioxidative properties, intracellular reactive oxygen species (ROS) levels were measured using the DCFDA dye. Piperine did not reduce the increase in intracellular ROS induced by either 6‐OHDA or H_2_O_2_ (Figure 5D,E). Additionally, the potential of piperine to enhance the expression of antioxidant‐related genes, including superoxide dismutase 1 (*SOD1*), catalase (*CAT*), and glutathione peroxidase 1 (*GPX1*) was evaluated. Following incubation with 20 μM piperine, there was no significant increase in the expression of these antioxidant‐related genes compared to the control group (Figure 5F).

In summary, these results suggest that piperine at a concentration of 20 μM mitigates the 6‐OHDA‐induced cytotoxicity by reducing mitochondrial superoxide levels. However, this effect does not appear to be mediated by enhanced antioxidative properties.

### 3.6 The Mechanism of Piperine in Modulating 6‐OHDA‐Induced Mitochondrial and Endoplasmic Reticulum STRESS

**FIGURE 6:**
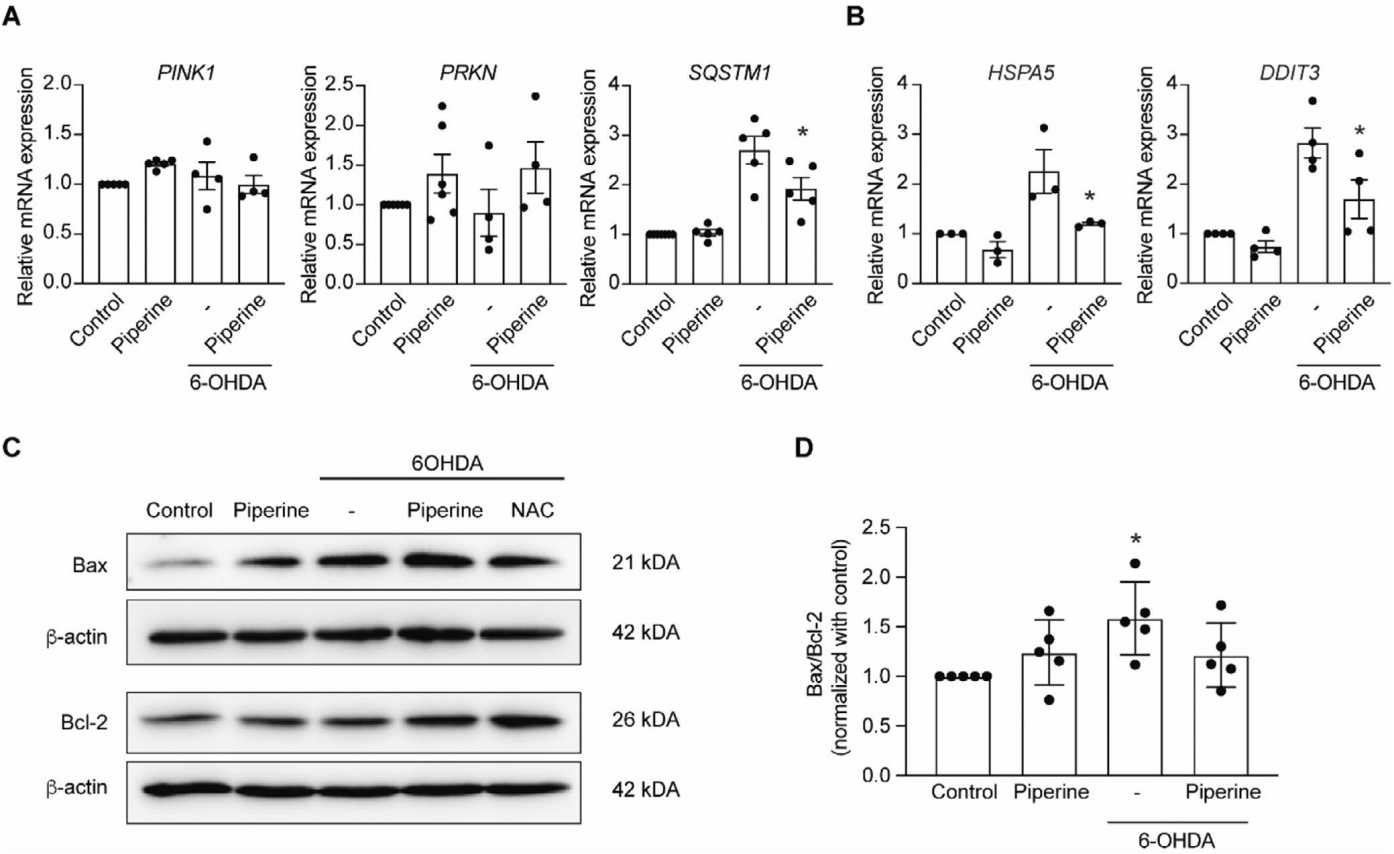
Piperine does not affect antioxidant properties in SH‐SY5Y cells. (A) Bar graphs representing the relative mRNA expression levels as a fold change compared to the control across independent experiments of *PINK1* (left), *PRKN* (middle) and *SQSTM1* (right) in control, 20 μM piperine, 6‐OHDA and 20 μM piperine +6‐OHDA treated group (one‐way ANOVA, *n* = 4–5 independent cell culture preparations). (B) Bar graphs representing the relative mRNA expression as a fold change compared to the control across independent experiments levels of *HSPA5* (left) and *DDIT3* (right) in control, 20 μM piperine, 6‐OHDA and 20 μM piperine +6‐OHDA treated group (*p < 0.05, compared with 6‐OHDA treated group, one‐way ANOVA, *n* = 3–4 independent cell culture preparations). (C) Representative of BAX, Bcl‐2 and β‐actin protein expression as a fold change compared to the control across independent experiments by Western blot analysis in control, 20 μM piperine, 6‐OHDA,6‐OHDA +20 μM piperine, 6‐OHDA + NAC. (D) Bar graph represents a normalized densitometric quantification of Bax to Bcl‐2 as a fold change compared to the control across independent experiments in control, 20 μM piperine, 6‐OHDA and 20 μM piperine +6‐OHDA treated group (*p < 0.05 compared with control group, one‐way ANOVA, *n* = 5 independent cell culture preparations).

We investigated the effect of piperine on the consequences of mitochondrial stress induced by 6‐OHDA. Mitochondrial quality control genes, including the sensors and effectors of mitochondrial damage, PTEN induced kinase 1 (*PINK1*) and parkin RBR E3 ubiquitin protein ligase (*PRKN*), (Narendra and Youle 2024) as well as sequestosome‐1 (*SQSTM1*), a key regulator of mitophagy in response to mitochondrial damage (Poon et al. 2021), were evaluated. The piperine‐treated group showed a trend toward increased expression of both *PINK1* and *PRKN* genes, but the changes did not reach statistical significance (Figure 6A). Interestingly, piperine significantly alleviated the increase in *SQSTM1*gene expression caused by 6‐OHDA (Figure 6A). Because mitochondrial stress can lead to oxidative stress and subsequent endoplasmic reticulum (ER) dysfunction via activation of the unfolded protein response protein binding immunoglobulin protein (BiP, encoded by *Homo sapiens* heat shock protein family A member 5 (*HSPA5*)) and transcription factor c/EBP‐homologous protein (CHOP, encoded by DNA Damage Inducible Transcript 3 (*DDIT3*)), ultimately leading to cell death (Yamamuro et al. 2006). Interestingly, piperine significantly reduced the 6‐OHDA‐induced increase in *HSPA5* and *DDIT3* gene expression (Figure 6B). In addition, we assessed the downstream consequences of mitochondrial and ER stress on apoptosis by evaluating the BAX/BCL‐2 ratio. Figure 6C reveals that piperine significantly reduced the BAX/BCL‐2 ratio in 6‐OHDA‐treated cells. These results suggest that piperine reduces 6‐OHDA‐induced mitochondrial and ER stress, thereby preventing the activation of the apoptosis pathway.

## 4 Discussion

**FIGURE 7:**
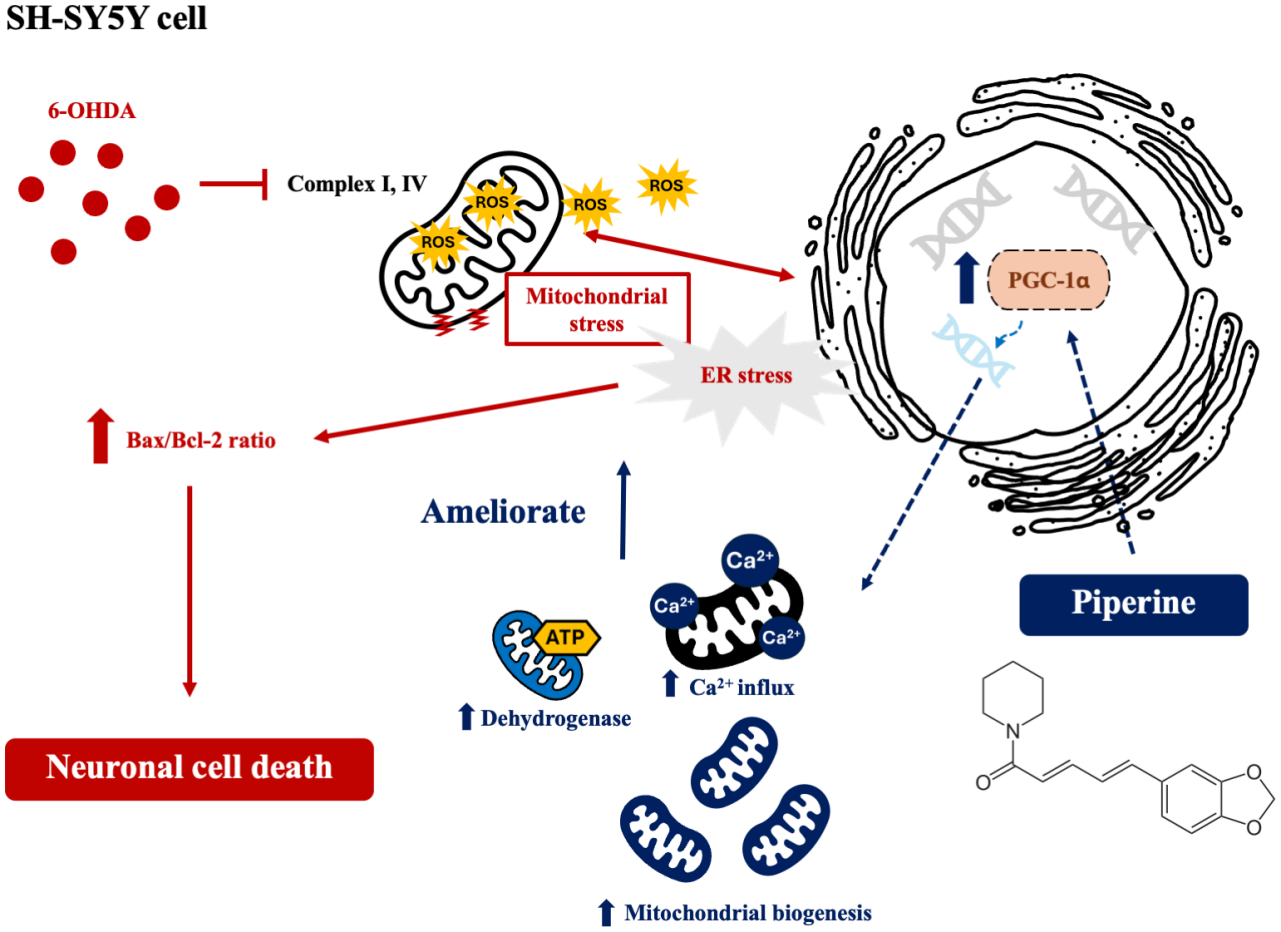
Mechanism by which piperine enhances mitochondrial biogenesis to ameliorate mitochondrial stress. A schematic illustrating piperine increases an expression mitochondrial biogenesis regulator PGC‐1α, leading to an increase in mitochondrial mass, enhanced mitochondrial Ca^2+^ influx and increased in mitochondrial metabolic enzyme. These effects ameliorate mitochondrial injury‐induced neuronal death caused by 6‐OHDA.

Piperine has been shown to exert several beneficial effects in both in vitro and in vivo studies. In the CNS, it has been reported to improve and prevent neuronal damage through various mechanisms, including antioxidant properties, promotion of autophagy, and mitochondrial protection (Azam et al. 2022; Yu et al. 2024). However, the cellular mechanisms by which piperine influences mitochondrial dynamics remain unclear. In this study, we investigate the effect of piperine on mitochondrial dynamics in a neuroblastoma cell line. Our findings provide evidence that piperine enhances mitochondrial biogenesis, leading to increased mitochondrial enzymatic function and improved calcium transport. These effects contribute to greater tolerance to mitochondrial stress induced by 6‐OHDA, resulting in a reduction of mitochondrial superoxide production. Furthermore, piperine reduces the activation of ER stress response genes *DDIT3* and *HSPA5*, leading to a decrease in apoptosis signaling, as indicated by a reduced BAX/Bcl‐2 ratio, and ultimately mitigates 6‐OHDA‐induced cell death (Figure 7).

To investigate the beneficial effects of piperine on neuronal cells, a well‐characterized human neuroblastoma cell line, SH‐SY5Y, was used in this study. During the initial toxicity testing of piperine on SH‐SY5Y cells, an increase in MTT assay activity was observed. This increase in MTT assay activity can be a result of either: (1) an increase in cell proliferation leading to a higher cell number or (2) an increase in mitochondrial enzyme activity and mitochondrial mass without a change in cell number. Our results suggest that the observed increase in MTT assay activity is most likely due to an increase in mitochondrial mass. The supporting evidence includes (1) piperine does not alter cell density and cell proliferation, (2) piperine increases the protein expression of TOM20, an outer mitochondrial membrane protein commonly used as a marker for mitochondrial abundance (Park et al. 2019). Additionally, an increase in the expression of *PPARGC1A*, which encodes PGC‐1α, a master regulator of mitochondrial biogenesis, further supports the role of piperine in promoting mitochondrial mass expansion. These findings indicate that piperine enhances both mitochondrial mass and mitochondrial dehydrogenase enzyme activity. This mechanism of mitochondrial biogenesis is essential for maintaining mitochondrial health and plays a crucial role in cellular recovery following mitochondrial injury. It has been shown that the downregulation or inhibition of PGC‐1α worsens pathology in vitro and in vivo models of liver fibrosis (Zhang et al. 2020) and exacerbates ethanol‐induced mitochondrial injury in PC12 cells (You et al. 2024). Our results are consistent with and provide additional support for previous studies showing that piperine prevents mitochondrial dysfunction in a rat model of ischemic brain injury (Kaushik et al. 2021) and diabetic cardiomyopathy (Wang et al. 2020).

Beyond mitochondrial biogenesis, mitochondrial dynamics, specifically fission and fusion processes, are crucial for maintaining the energy balance within cells (Chen et al. 2023; Sukhorukov et al. 2024). Mitochondrial fission is primarily regulated by the DRP1 protein, while fusion is controlled by mitofusin and OPA1 protein (Giacomello et al. 2020). Previous studies have suggested that piperine may activate mitofusin function and promote mitochondrial elongation in murine embryonic fibroblasts (Zhang et al. 2022). In our study, although piperine increases mitochondrial mass and causes a slight reduction in the proportion of very long mitochondria, the overall mitochondrial morphology remains largely unchanged. Additionally, piperine shows a trend toward increasing OPA1 and MFN2 protein expression; however, these changes do not reach statistical significance.

In addition to mitochondrial dynamics, the concentration of Ca^2+^ in mitochondria plays a critical role in regulating mitochondrial respiration and ROS‐generating enzymes (McCormack and Denton 1990; Wang et al. 2013). A decrease in mitochondrial Ca^2+^ concentration can lead to a reduction in mitochondrial membrane potential and impaired oxidative phosphorylation (OXPHOS) (Raby et al. 2024). Our analysis of mitochondrial Ca^2+^ revealed that piperine enhances mitochondrial Ca^2+^ flux, which aligns with our previous finding that piperine promotes mitochondrial mass and mitochondrial enzymatic activity.

It is widely recognized that mitochondrial dysfunction plays a key role in the pathogenesis of several neurodegenerative diseases, including Parkinson's disease. Our results demonstrate that enhancing mitochondrial properties with piperine can protect mitochondria from damage caused by 6‐OHDA, a catecholaminergic neurotoxicant commonly used as an in vitro model of Parkinson's disease (Simola et al. 2007; Xicoy et al. 2017). These findings are consistent with previous reports in both in vitro and in vivo models (Bi et al. 2015; Liu et al. 2016; Yu et al. 2024). Although previous studies suggest that the neuroprotective mechanisms of piperine involve its antioxidative properties (Bi et al. 2015; Tripathi et al. 2022) or an enhancement of autophagic activity to remove damaged mitochondria (Liu et al. 2016; Yu et al. 2024), our results reveal an additional dimension of piperine's neuroprotective effect: its ability to enhance mitochondrial function independently of antioxidative pathways (Figures 4 and 5). This effect prevents the activation of mitophagy and ER stress, which are consequences of mitochondrial dysfunction, ultimately inhibiting downstream apoptosis signaling and cell death. However, the potential protective effects of piperine under broader oxidative stress conditions may require further investigation using alternative concentrations or experimental models to better elucidate its role in oxidative stress regulation. Notably, piperine has also been reported to modulate intracellular calcium homeostasis by influencing both extracellular calcium influx and ER calcium handling (Hsieh et al. 2019; Zhang et al. 2013). This may, in part, explain the observed reduction in ER stress markers following piperine treatment. Nevertheless, further investigation is needed to fully elucidate the role of calcium regulation in piperine's protective mechanism. Above all, our results highlight the therapeutic potential of piperine for mitigating mitochondrial damage, particularly in Parkinson's disease. However, since the CNS is composed of multiple cell types that may respond differently to piperine, further studies are needed using complex models, such as iPSC‐derived neuronal cells or in vivo systems, to better understand the heterogeneity of cell responses and to recapitulate the effects of piperine in the brain.

## 5 Conclusion

In conclusion, this study demonstrates that piperine enhances mitochondrial function by increasing mitochondrial biogenesis and enzymatic activity. These effects mitigate mitochondrial injury caused by 6‐OHDA‐induced stress. Additionally, piperine prevents mitophagy, ER stress, and Bax/Bcl‐2 activation following mitochondrial stress. Further studies are required to explore the protective mechanisms of piperine in other cell types or disease models, which could underscore its potential benefits in addressing mitochondrial dysfunction.

## Author Contributions

**Nongluk Saikachain:** formal analysis (equal), investigation (equal), methodology (equal). **Pawitchaya Charoensawat:** formal analysis (equal), investigation (equal), visualization (equal), writing – original draft (equal), writing – review and editing (equal). **Oramon Tuntoolavest:** formal analysis (equal), investigation (equal), writing – review and editing (supporting). **Napak Vejsureeyakul:** formal analysis (equal), investigation (equal), writing – review and editing (supporting). **Teeranart Kunpittaya:** formal analysis (equal), investigation (equal), writing – review and editing (supporting). **Wanangkan Poolsri:** investigation (supporting). **Titiwat Sungkaworn:** conceptualization (equal), data curation (equal), formal analysis (equal), writing – review and editing (equal). **Rungnapha Saeeng:** resources (equal), supervision (equal), writing – review and editing (equal). **Chatchai Muanprasat:** conceptualization (equal), data curation (equal), formal analysis (equal), funding acquisition (equal), project administration (equal), resources (equal), supervision (equal), writing – review and editing (equal). **Nithi Asavapanumas:** conceptualization (equal), data curation (equal), formal analysis (equal), investigation (equal), methodology (equal), validation (equal), visualization (equal), writing – original draft (equal), writing – review and editing (equal).

## Conflicts of Interest

The authors declare no conflicts of interest.

## Supporting information


Data S1.

Data S2.

Data S3.

Data S4.

## Data Availability

All relevant data are included in the manuscript. Additional raw data are available from the corresponding author upon request.
