# Supplementary material for: Piperine Enhances Mitochondrial Biogenesis to Mitigate Stress in SH‐SY5Y Neuroblastoma Cells
Source: Food Sci Nutr. 2025 Jul 16;13(7):e70637. doi: 10.1002/fsn3.70637 (PMC12267666; doi:10.1002/fsn3.70637)
Supplement: Supplementary file 1 — Data S1. [file FSN3-13-e70637-s004.pptx]

## Slide 1
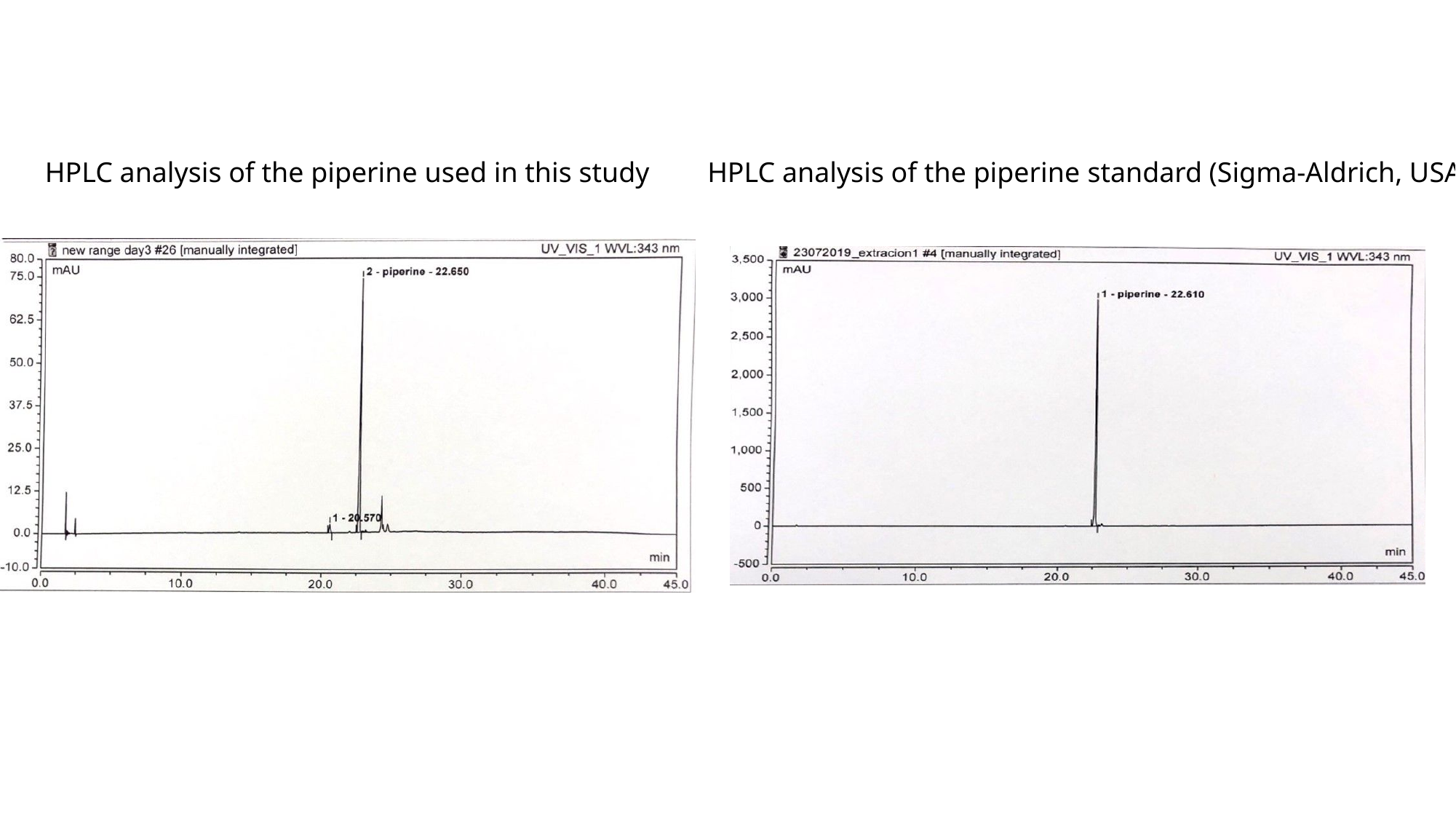

HPLC analysis of the piperine used in this study
HPLC analysis of the piperine standard (Sigma-Aldrich, USA)
