# Supplementary material for: Piperine Enhances Mitochondrial Biogenesis to Mitigate Stress in SH‐SY5Y Neuroblastoma Cells
Source: Food Sci Nutr. 2025 Jul 16;13(7):e70637. doi: 10.1002/fsn3.70637 (PMC12267666; doi:10.1002/fsn3.70637)
Supplement: Supplementary file 2 — Data S2. [file FSN3-13-e70637-s001.docx]

**Supporting Information S2. Dose–response effect of hydrogen peroxide (H₂O₂) on SH-SY5Y cell viability.** SH-SY5Y cells were treated with various concentrations of H₂O₂ for 24 hours. Cell viability was assessed using the MTT assay and expressed as a percentage relative to untreated control cells. n = 3 independent experiments. The IC₅₀ value was approximately 105.5 μM.
