# Supplementary material for: Piperine Enhances Mitochondrial Biogenesis to Mitigate Stress in SH‐SY5Y Neuroblastoma Cells
Source: Food Sci Nutr. 2025 Jul 16;13(7):e70637. doi: 10.1002/fsn3.70637 (PMC12267666; doi:10.1002/fsn3.70637)
Supplement: Supplementary file 3 — Data S3. [file FSN3-13-e70637-s003.docx]

**Supporting Information S3. Effect of piperine on SH-SY5Y cell viability.** Bar graph represents the percentage of SHSY-5Y cell viability compared to the control after incubating with piperine at the concentrations ranging from 1.25 to 80 mM for 24 h across independent experiments. * P< 0.05 compared with the control group (one-way ANOVA, n = 6 independent cell culture preparations).
