# Supplementary material for: Piperine Enhances Mitochondrial Biogenesis to Mitigate Stress in SH‐SY5Y Neuroblastoma Cells
Source: Food Sci Nutr. 2025 Jul 16;13(7):e70637. doi: 10.1002/fsn3.70637 (PMC12267666; doi:10.1002/fsn3.70637)
Supplement: Supplementary file 4 — Data S4. [file FSN3-13-e70637-s002.docx]

**Supporting Information S4.** **Piperine protects SH-SY5Y cells against 6-OHDA-induced cytotoxicity.** Bar graph represents the percentage of SHSY-5Y cell viability compared to the control across independent experiments in the following groups: control, 6-OHDA, 6-OHDA with piperine at concentrations ranging from 5 to 80 uM, and 6-OHDA + NAC groups. * P<0.05 compared with 6-OHDA group, one-way ANOVA, n= 5 independent cell culture preparations.
